# Case Report: Clinical and Serological Hallmarks of Cytokine Release Syndrome in a Canine B Cell Lymphoma Patient Treated With Autologous CAR-T Cells

**DOI:** 10.3389/fvets.2022.824982

**Published:** 2022-07-11

**Authors:** Matthew J. Atherton, Antonia Rotolo, Kumudhini P. Haran, Nicola J. Mason

**Affiliations:** ^1^Department of Clinical Sciences and Advanced Medicine, School of Veterinary Medicine, University of Pennsylvania, Philadelphia, PA, United States; ^2^Department of Biomedical Sciences, School of Veterinary Medicine, University of Pennsylvania, Philadelphia, PA, United States; ^3^Parker Institute for Cancer Immunotherapy, University of Pennsylvania, Philadelphia, PA, United States; ^4^Department of Pathobiology, School of Veterinary Medicine, University of Pennsylvania, Philadelphia, PA, United States

**Keywords:** CAR-T cells, cytokine release syndrome (CRS), canine B cell lymphoma, comparative oncology, spontaneous tumor model

## Abstract

**Background:**

Chimeric antigen receptor-T (CAR-T) cells have transformed the treatment of human B cell malignancies. With the advent of CAR-T therapy, specific and in some cases severe toxicities have been documented with cytokine release syndrome (CRS) being the most frequently reported. As dogs develop tumors spontaneously and in an immunocompetent setting, they provide a unique translational opportunity to further investigate the activity and toxicities associated with CAR-T therapy. Although various adoptive cellular therapy (ACT) trials have been documented and several more are ongoing in canine oncology, CRS has not been comprehensively described in canine cancer patients.

**Case Presentation:**

Here we present the clinical and serologic changes in a dog treated with autologous CAR-T for relapsed B cell lymphoma that presented with lethargy and fever 3 days following CAR-T. Multiplexed serum cytokine profiling revealed increases in key cytokines implicated in human CRS including IL-6, MCP-1, IFNγ and IL-10 at or shortly after peak CAR-T levels *in vivo*.

**Conclusion:**

The observations noted in this case report are consistent with CRS development following CAR-T therapy in a canine patient. The dog represents a compelling model to study the pathophysiology of CRS and pre-clinically screen novel therapeutics to prevent and treat this life-threatening condition in the setting of a complex and naturally evolved immune system.

## Introduction

Adoptive cellular therapy (ACT) has transformed the treatment of certain cancers including B cell malignancies. ACT utilizing T cells involves the isolation, *ex vivo* expansion (with or without engineering to redirect T cell specificity) and re-infusion of T cells into the patient (1). T cell specificity can be redirected using two well described methods (1), i.e., either a desired T cell receptor (TCR) is selected and cloned into T cells, or an antigen-recognizing antibody fragment is coupled to T cell linkers and signaling domains resulting in a synthetic chimeric antigen receptor (CAR). Responses in refractory B cell neoplasms treated with CAR-T cells have been particularly impressive (2, 3), including durable complete responses in patients with otherwise lethal B cell malignancy and with no other therapeutic options (1). Aligned with these successes, anti-CD19 CAR-T cells have received FDA approval for relapsed/ refractory precursor acute lymphoblastic leukemia (ALL) and diffuse large B cell lymphoma (DLBCL) (4). CAR-T therapy is still in its infancy in veterinary medicine, however, owing to the curative potential and high translational relevance of canine CAR-T research, this field is growing rapidly. Dogs develop spontaneous B cell lymphomas with clinical and biological features that mirror those of their human counterparts, thus representing a very attractive parallel patient population in which to perform clinically relevant translational studies. Initial trials using CD20-directed CAR-T cells in dogs diagnosed with B cell lymphoma revealed the feasibility of this approach with early signs of activity and documented cases of target antigen down-regulation (5, 6).

Although clinical response rates of CAR-T are remarkable, novel and substantial toxicities have also been noted in human medicine. Two major manifestations of post-infusion CAR-T toxicity are cytokine release syndrome (CRS) and neurotoxicity, with toxicities ranging from mild flu-like illness to being rapidly fatal (1, 3, 7). CRS is usually encountered within days to the first 2 weeks of CD19-specific CAR-T cell infusion and occurs at the time of peak CAR-T expansion *in vivo* (1, 7, 8). Clinical signs include chills, myalgia, fever and in more severe cases hypotension and respiratory compromise (1, 8). IL-6, IL-10 and IFNγ are amongst the most significantly elevated cytokines following CART-19 treatment in people (8). While CRS has been extensively studied and seems to correlate with tumor burden at the time of CAR-T cell infusion and the dose of CAR-T cell administered, the development of neurotoxicities post CAR-T is not as predictable (1, 3, 9). Potential explanations for the latter toxicity include cytokine-induced endothelial injury in neurological tissue as well as antigen expression (e.g., CD19) by mural cells within the brain (3, 10).

CART-19 trials have shown that CRS affects upwards of 77% of patients with recent data showing that up to 7% of patients will suffer from non-relapse mortality in the first 30 days following CAR-T therapy. As such, there is an urgent need to develop model systems to aid our understanding of this condition and provide opportunities to improve the clinical management of CRS (11–14). To this end both murine and non-human primate systems have been developed to recapitulate CRS. Two murine models of CRS utilizing transplantable tumors have been described and utilize SCID and humanized mice to mimic the clinical occurrence of CRS (15, 16). Neurotoxicity and CRS has also been described following infusion of autologous CART-20 cells in immune competent tumor free non-human primates (NHP) (17). Although these studies have provided valuable data regarding CRS, they are limited by the fact that tumors are either transplanted into a xenogeneic host or are not present at all. Therefore, the natural steps of cancer immunoevasion are absent and the interaction between CAR-T and other key immune effectors, including antigen expressing target cells, does not completely recapitulate clinical CRS. Models that spontaneously develop tumors and exhibit cancer associated immune dysfunction are predicted to more accurately parallel CRS in human cancer patients following CAR-T administration, than other experimental models. To this end, dogs that develop spontaneous cancers are being increasingly employed to evaluate safety and efficacy of next generation CAR-T cell therapies (18, 19). As client owned dogs begin to receive CAR-T infusions, the documentation of adverse events secondary to such treatment appears inevitable. Thus, they are uniquely positioned to further our mechanistic understanding of CRS and provide a platform to assess novel interventions to treat this syndrome.

In the first report of therapeutic use of permanently transduced CD20-targeted CAR-T cells administered to pet dogs with spontaneous B-NHL, a favorable early safety profile was observed in the five treated dogs with no CAR-T specific adverse events recorded (6). In this case report, we describe the presentation of a canine patient treated with autologous lentiviral redirected anti-CD20 CAR-T cells developing clinical signs consistent with CRS in the context of multiplexed serum cytokine analysis and quantitative CAR-T cell kinetic profiling. These findings suggest that pet dogs with spontaneous hematological malignancies will serve as valuable models to investigate CAR-T associated CRS and therapeutic strategies to reduce its occurrence.

## Case Presentation

A 9-year-old male castrated Pembrokeshire Welsh corgi presented to the Matthew J. Ryan Veterinary Hospital of the University of Pennsylvania (MJR-VHUP) for assessment of relapsed multicentric lymphoma. The patient was initially diagnosed cytologically with large B-cell lymphoma of at least stage IIIa, 6 months prior he had attained an initial complete response following an abbreviated CHOP-based protocol. Complete staging at MJR-VHUP was consistent with stage IVa B cell lymphoma and flow cytometry of a peripheral nodal aspirate revealed the neoplastic B cells to be positive for CD20 and CD79a. Due to the patient's small size, apheresis was not performed to collect peripheral blood mononuclear cells (PBMCs) for product generation. Instead, at the time of staging ~45 mls of whole blood was collected in citrate, PBMCs were isolated using Ficoll-Paque PLUS (GE Healthcare) and antibody-based negative selection of canine T cells was performed as previously described (6). T cells were then cryopreserved in liquid nitrogen as the patient's owner elected to pursue conventional chemotherapeutic rescue (rabacfosadine, Tanovea-CA1, VetDC) prior to CAR-T therapy.

**Figure 1:**
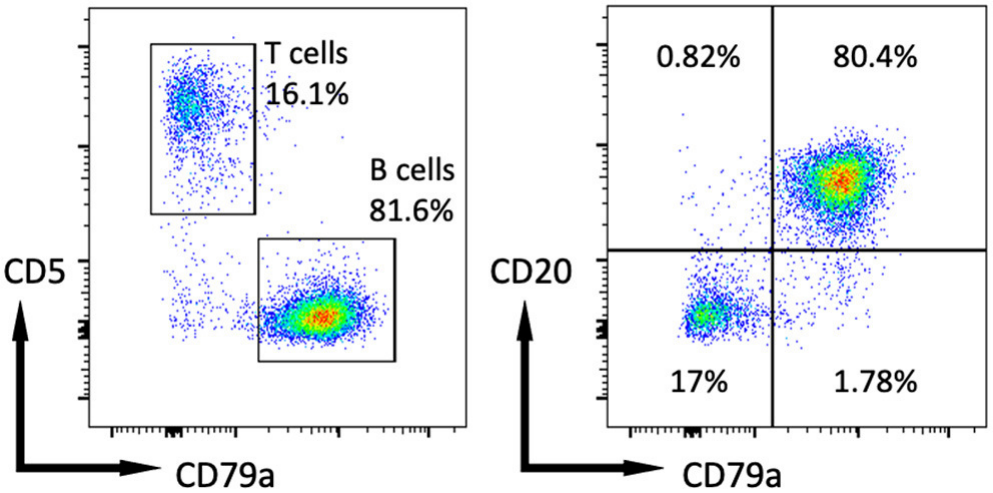
Flow cytometry of cells isolated from fine needle aspiration of the patient's left prescapular lymph node and stained for CD5-PE, CD79a-APC and CD20-BV421 as previously described (6).

**Figure 2:**
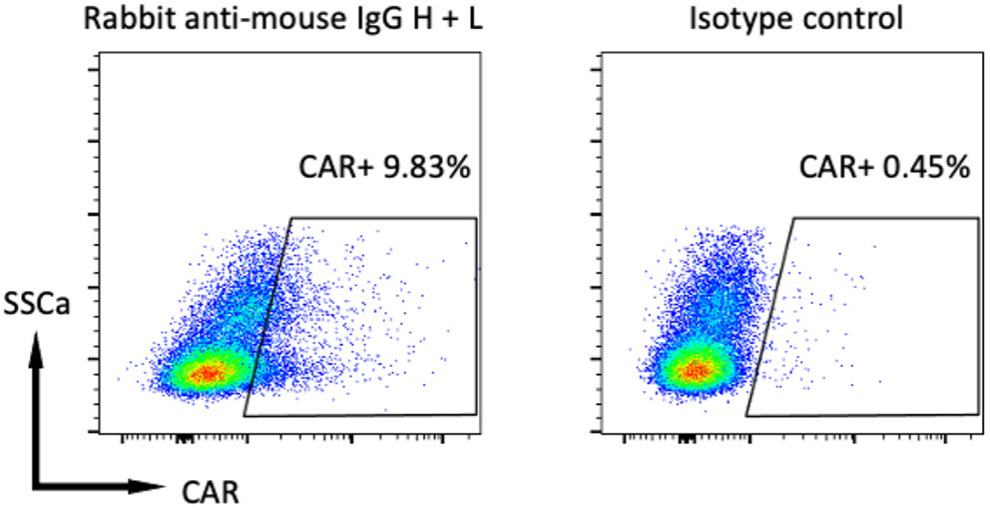
Flow cytometry of CAR-T cell product 12 days after T cell activation. Gated on live, CD5+ cells that were stained for CAR using a biotinylated rabbit anti-mouse IgG H + L antibody and isotype control antibody with a secondary APC-streptavidin conjugate as previously described (6).

The patient attained a complete response following rabacfosadine treatment which lasted ~6 months prior to second relapse. Flow cytometry at this time demonstrated preserved CD20 expression by the neoplastic cells (Figure 1), confirming eligibility for autologous CD20-specific CAR-T cell therapy. Therefore, the patient's cryopreserved T cells were thawed and activated in T cell media supplemented with 100 U/ml rhIL-2 (Gibco) and 10 ng/ml rhIL-21 (eBioscience) using agonistic anti-canine CD3 (clone CA17.2A12, BioRad) and CD28 (clone 1C6, eBioscience) antibodies conjugated to magnetic tosylactivated magnetic Dynabeads (at a ratio of 3 beads: 1 T cell) manufactured as previously described (5). T cells were activated in the presence of 1 μM ralimetinib dimesylate (MedChemExpress), a p38α and p38β mitogen-activated protein kinase (MAPK) inhibitor based on preclinical studies showing p38 inhibition to enhance CAR-T function both *in vitro* and *in vivo* (20, 21). Twenty four hours after activation, T cells were transduced with cCD20-BB-ζ lentivirus (6) at a multiplicity of infection of 10. Cells were supplemented again with 100 U/ml rhIL-2 and 10 ng/ml rhIL-21 3 days post activation, then switched to supplementation with 20 ng/mL rhIL-7 (Peprotech) and 10 ng/mL rhIL-15 (Peprotech) on days 5, 8, 11, and 13 post-activation. All T cells were re-stimulated at a ratio of ~8.5 T cells:1 irradiated CD20+ CLBL-1 cells [initially established by Rütgen et al. (22)] on day 11 post-activation and 50% of the cells were retreated with 1μM ralimetinib dimesylate day 13 post-activation. At day 15 post-activation magnetic beads were removed from the T cells, cells were washed and resuspended in Plasma-Lyte A (Baxter). Transduction was quantified flow cytometrically (Figure 2) using previously described protocols (6) and a dose of ~5 ×10^6^ CAR-T/kg (a total of ~50 ×10^6^ total T cells/kg) of patient's body weight was attained, this represented a ten-fold increase in CAR-T dose compared to our previously published study (6). Standard QC tests for sterility and safety (6, 23) on a sample from the final CAR-T cell product confirmed the lack of bacterial and mycoplasma contamination. No Replication Competent Lentivirus was detected (24).

**Figure 3:**
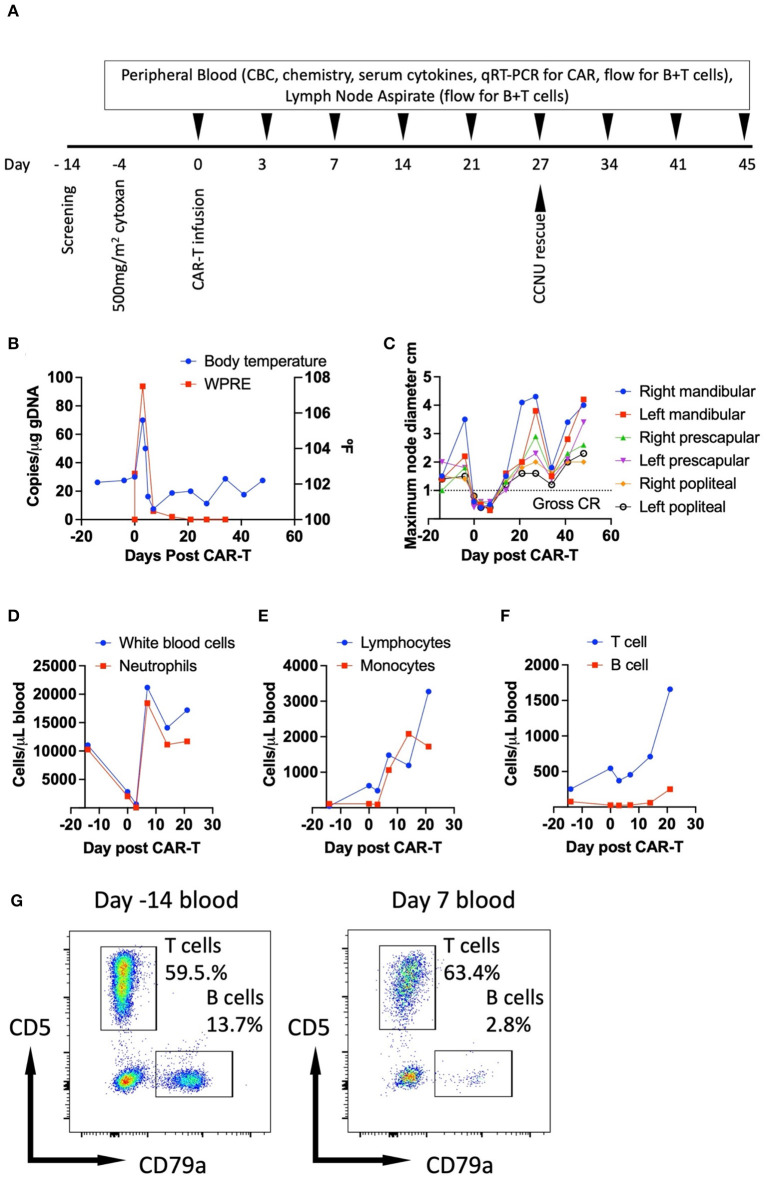
Clinical summary of patient's response to CAR-T therapy. Therapeutic timeline **(A)**; monitoring of body temperature and *in vivo* CAR-T cell levels **(B)**; tracking of target node dimensions **(C)**; circulating white blood cell and neutrophil counts **(D)**; circulating lymphocyte and monocyte counts **(E)**; tracking of circulating T and B cell counts determined by the product of the frequencies CD5+ or CD79a+ live cells in the lymphogate quantified flow cytometrically, and the number of circulating lymphocytes obtained by complete blood count **(F)** and flow cytometry performed on PBMCs stained for CD5-PE and CD79a-APC at day−14 and day 7 post-CAR-T **(G)**. WPRE- woodchuck hepatitis virus post-transcriptional regulatory element, used for detection of genomic copies of CAR by qRT-PCR as previously described (6); CR- complete response.

Four days prior to CAR-T cell infusion the patient received 500 mg/m^2^ cyclophosphamide (Baxter) intravenously for lymphodepletion (Figure 3A). For prophylaxis against sterile hemorrhagic cystitis, intravenous fluid therapy was prescribed for 24 h, and a single dose of 3 mg/kg furosemide (Merck Animal Health) was administered concurrently with cyclophosphamide and 5 doses of 200 mg/m^2^ mesna (Baxter) were administered intravenously every 3 h. The patient was discharged with 2 weeks of prophylactic enrofloxacin (Bayer) and 5 days of furosemide both given by mouth. On the day of CAR-T cell infusion (Figure 3A) the patient received intramuscular diphenhydramine (Hikma) (2 mg/kg) and intravenous ondansetron (West Ward) (0.2 mg/kg) premedication followed by slow intravenous CAR-T cell infusion. The patient tolerated the infusion well and remained hemodynamically stable throughout the infusion and during the post-infusion observation period after which he was discharged for planned routine follow up (Figure 3A).

On the third morning following CAR-T therapy, the patient presented as an emergency to MJR-VHUP for inappetence and lethargy with the owner documenting a temperature of 104.3°F. Physical examination confirmed pyrexia at 105.6°F (Figure 3B), and a moderate tachycardia (160 BPM) with accompanying hyperdynamic pulses noted. Lymph nodes were small and soft on palpation (Figure 3C). Other findings included a grade II/VI bilateral systolic murmur and a right corneal descemetocele, these latter two findings were recorded on prior exams and were deemed stable. Abdominal palpation was normal and not resented. The patient was subsequently hospitalized within the ICU and all applicable toxicities were graded according to the VCOG-CTCAE v2 scheme (25). A grade 1 systolic hypertension of 180 mmHg was documented shortly after admission. Complete blood counts revealed grade 1 non/pre-regenerative anemia (HCT 39.7%), a grade 4 neutropenia (0.03 ×10^3^/μL), a grade 2 thrombocytopenia (53 × ^3^/μL), a moderate lymphopenia (0.48 × ^3^/μL), a monocytopenia (0.09 × ^3^/μL) and eosinopenia (0.01 × ^3^/μL) (Figures 3D,E) (25). These findings were consistent with the prior administration of high dose cyclophosphamide. Serum chemistry revealed no significant changes except for hypomagnesemia (1.2 mg/dL) which was considered most likely to be spurious due to slight sample hemolysis but an iatrogenic origin secondary to furosemide administration could not be entirely excluded. Urinalysis revealed moderate hematuria and proteinuria, white blood cells were sparsely present, and no infectious agents were observed. Although no obvious nidus of infection was observed at admission the patient's antibiotic coverage was changed to intravenous piperacillin and tazobactam (Sandoz) owing to the degree of neutropenia and supportive crystalloid IVFT was administered for a presumptive diagnosis of grade 2 CRS based on the presence of a fever and the need for hospitalization for supportive care (25). Over the following 36 h the patient remained hemodynamically stable however the systolic murmur increased in intensity and echocardiography revealed stage B1 degenerative disease affecting the mitral and tricuspid valves with an abnormal relaxation pattern observed likely secondary to hypertension. No vegetative lesions were detected. The patient was discharged at day 5 post-CAR-T as he was no longer inappetent nor lethargic, the pyrexia, tachycardia, leukopenia and hypomagnesemia had resolved. However, the patient remained anemic and thrombocytopenic without evidence of spontaneous hemorrhage. The patient was prescribed 2 weeks of oral clavulanate-potentiated amoxicillin (Zoetis). Both the red cell and platelet counts normalized over the following 2 weeks, body temperature (Figure 3B) and hemodynamic status remained within normal limits. The patient's lymph nodes were noted to be mildly enlarged 14 days post-CAR-T and this was progressive leading to the prescription of a CCNU based rescue regimen 27 days post-CAR-T (Figures 3A,C). A non-durable partial response to the rescue regimen was documented (Figure 3C). However, the owner elected for euthanasia at day 73 post-CAR-T for progressive disease. A necropsy was not performed.

**Figure 4:**
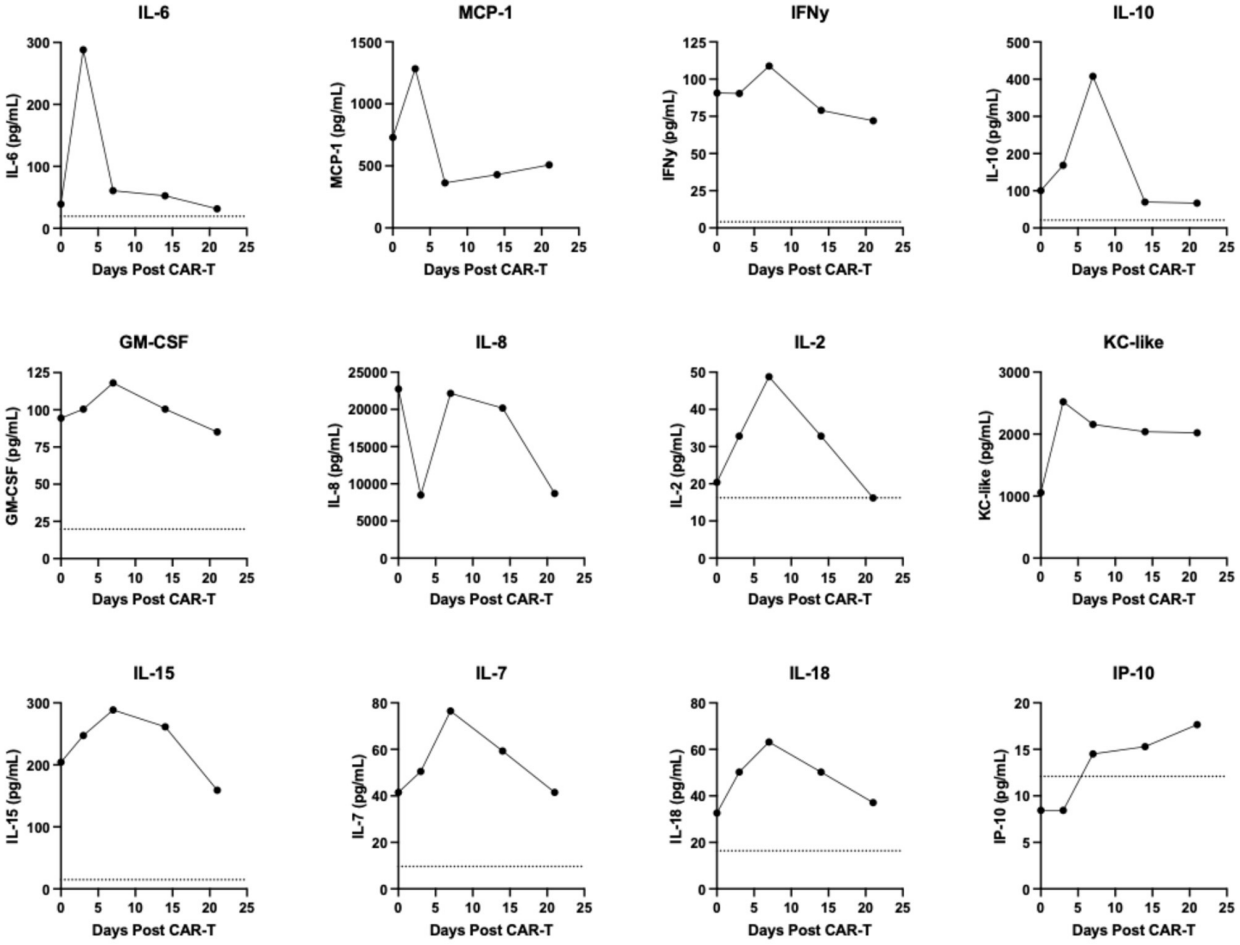
Serial multiplexed analysis of serum cytokines. Dashed lines represent lower limits of detection, dashed lines are superimposed with *x*-axes for those plots where they are not visible.

Retrospective ancillary diagnostic testing was undertaken to investigate the etiology of the pyrexia, lethargy, and cardiovascular changes. Flow cytometry performed on peripheral blood as described previously (6) revealed a depletion of circulating B cells temporally coinciding with normal peripheral nodal measurements (Figures 3C,F,G). At disease relapse numbers of both circulating B and T cells increased (Figure 3F). Circulating CAR-T cell kinetics were tracked using qRT-PCR as previously described (5). Notably CAR-T cell levels peaked 3 days post-CAR-T administration and at the time of documentation of pyrexia (Figure 3B). Following peak peripheral CAR-T cell signal at day 3, circulating CAR-T cell numbers contracted and were below the limit of detection on day 14 at which point the lymph nodes were increasing in dimension consistent with relapse (Figures 3B,C). Serum cytokines were run retrospectively and in duplicate for each of the indicated timepoints in a single batch using a commercially available multiplexed assay (MILLIPLEX^®^ Canine Cytokine Panel). Serum IL-6, MCP-1 and KC-like all increased and peaked in concentration at day 3 post-CAR-T cell administration prior to decreasing to varying degrees (Figure 4). Serum IFNγ, IL-10, GM-CSF, IL-2, IL-15, IL-7, and IL-18 all increased and peaked in concentration at day 7 post-CAR-T prior to decreasing (Figure 4). Serum IL-8 initially decreased at day 3 following CAR-T prior to returning to pre-CAR-T concentration and subsequently contracting (Figure 4). Serum IP-10 was below the limit of detection prior to CAR-T and was then detected just above the limit of detection from day 7 onwards (Figure 4) and TNFα was below the limit of detection at all time points (data not shown). Importantly, all changes in serum cytokine levels occurred at the time of, or immediately after peak CAR-T levels in the blood and coincided with B cell depletion and normal peripheral nodal diameters. Collectively, these findings, together with the clinical signs exhibited by the patient at day 3 post-CAR-T infusion, were consistent with grade 2 CRS as per the VCOG-CTCAE v2 scheme (25).

## Discussion

In this case report we provide a comprehensive clinical and serologic description of CRS following autologous CAR-T cell therapy for a dog diagnosed with relapsed high grade B cell lymphoma. Although CRS has been documented in people, mice and NHP following CAR-T cell infusion (8, 9, 15–17, 26), CRS has not been described in veterinary patients undergoing CAR-T therapy for the treatment of spontaneous malignancies. Characterizing CRS in veterinary patients has the potential to be highly translationally relevant for the understanding of this significant adverse event in human oncology and for evaluating strategies to prevent its occurrence without incurring the need for artificial and invasive NHP or mouse based models that are currently used (14–17, 26).

Analysis of serum cytokines in our canine patient revealed many similarities with the profile of CRS in humans. Notably we saw rapid elevations in IL-6, MCP-1, IFNγ and IL-10, which have been implicated in CRS following autologous CAR-T therapy in human patients by various groups (8, 9, 26). These four cytokines peaked either concurrently or just following the point of maximum CAR-T cell levels *in vivo*. Importantly IL-6 levels have been shown to peak at the time of maximal T cell expansion in humans (8) in alignment with our own observations. One notable difference between this patient and what is commonly described in humans and NHPs following CAR-T cell administration was the initial decline in serum IL-8, as typically this cytokine increases following infusion (9, 17). Future studies will help define whether this observation was patient specific or if this represents a repeatable finding. Although the reported serum cytokine profile and kinetics in relation to CAR-T levels support *bona fide* CRS, the presence of an opportunistic infection in a neutropenic patient cannot be entirely excluded. However, an obvious nidus for infection or biochemical parameters that would support a diagnosis of sepsis could not be found and marked neutropenia following pre-conditioning that occurs over the duration of peak fever is frequently observed in human CRS following autologous CAR-T cell treatment (9). Collectively, these findings support our diagnosis of CRS secondary to CAR-T cell infusion in this patient.

In this report the CRS was designated as grade 2 and responded to supportive care. In human oncology mild cases of CRS can be managed similarly. However, severe cases require more aggressive interventions including vasopressors, respiratory support, corticosteroids and tocilizumab, with the latter being a monoclonal antibody raised against the IL-6 receptor (8, 9, 26). As CRS in this dog was mild, tocilizumab was not indicated, and currently there are no reports of cross reactivity with canine IL-6 receptor, so it is possible that canine specific IL-6 directed therapies will be required for more severe cases of CRS in dogs. In humans, the severity of CRS has been found to correlate with peak CD8+ CAR-T cell expansion and this expansion was also correlated to CAR-T cell efficacy (26). Here, the CAR-T cells peaked rapidly at modest levels post-infusion and did not persist beyond 2 weeks. Loss of CAR-T cell signal was concurrent with peripheral nodal enlargement around 2 weeks post-CAR-T therapy. Our group has recently implemented a method to transduce canine T cells with greater efficiency (23) and this represents an improvement over the CAR-T cell manufacturing process used for the patient described here. As we work toward optimizing canine CAR-T cell manufacture and preconditioning regimens, we anticipate that the efficacy of autologous CAR-T cells will improve, and we will encounter high grade CRS in veterinary patients. Similarly as the occurrence of CRS in people is best described following anti-CD19 CAR-T therapy (8, 9, 26), treatment with anti-CD19 CAR-T instead of anti-CD20 CAR-T cells in dogs (27) is also likely to precede more frequent and severe documentation of CRS in canine CAR-T patients.

Encountering CRS in veterinary patients provides an opportunity to appraise novel therapeutic strategies to prevent or treat CRS in a clinically relevant model with spontaneous and aggressive B cell malignancies. Recent evidence reveals that despite the currently available treatment options for CRS a significant number of cancer patients experiencing CRS die soon after CAR-T cell administration (13). Because of this, various groups are working on different strategies to reduce the incidence and severity of CRS. Many promising new approaches to mitigate CRS have recently been reviewed, including GM-CSF depletion, JAK/STAT inhibition and transient suppression of CAR-T cell activation with dasatinib amongst others (14). Preclinical data generated from testing such novel therapeutic approaches in dogs could streamline the design of early human trials with prioritization based upon the activity and safety profiles of these next-generation interventions observed in canine cancer patients receiving CAR-T cell therapy.

## Conclusions

This case report describes the clinical, hematologic, and serologic changes consistent with CRS in a dog diagnosed with relapsed B cell lymphoma following CAR-T infusion. Human immune-oncology trials have documented variable presentations and severities of CRS with some known predictive factors being described. This patient had mild changes that resolved following supportive care and bore remarkable clinical and serological similarities to low grade CRS encountered in people post CAR-T. At the time of the febrile episode, it should be noted that as in people with CRS following a lymphodepleting preconditioning regimen and CAR-T therapy, the dog was markedly neutropenic. As such an undetected opportunistic infection could not be entirely excluded as contributing to the clinical and serologic changes documented. As CAR-T technologies improve for veterinary oncology patients the likelihood of encountering severe CAR-T associated AEs will greatly increase. Developing appropriate strategies for the successful management of CRS in dogs following CAR-T provides a unique platform to further our understanding of this syndrome. Preclinical screening of next generation strategies designed to ameliorate CRS, in an immune competent setting utilizing CAR-T cells for the treatment of spontaneous and aggressive B cell malignancies has significant potential to improve outcomes in patients diagnosed with this frequent and potentially deadly adverse event.

## Data Availability Statement

The raw data supporting the conclusions of this article will be made available by the authors, without undue reservation.

## Ethics Statement

The animal study was reviewed and approved by the University of Pennsylvania's Institutional Animal Care and Use Committee. Written informed consent was obtained from the owners for the participation of their animals in this study.

## Author Contributions

MA, AR, and NM wrote the manuscript. MA and NM collected the patient's medical records. MA, AR, and KH performed laboratory tests and interpreted data. All authors contributed to the article and approved the submitted version.

## Funding

MA is supported by NIH/NCI K08CA252619. AR is supported by the ASGCT and the NIH U24-CA224122 award. NM is supported by NIH/NIAMS R01AR075337, NIH/NCI U24CA224122, and NIH/NCI U54 CA244711.

## Conflict of Interest

The authors declare that the research was conducted in the absence of any commercial or financial relationships that could be construed as a potential conflict of interest.

## Publisher's Note

All claims expressed in this article are solely those of the authors and do not necessarily represent those of their affiliated organizations, or those of the publisher, the editors and the reviewers. Any product that may be evaluated in this article, or claim that may be made by its manufacturer, is not guaranteed or endorsed by the publisher.
